# Whole Transcriptomic Profiling in Metastatic Liver Cancer With Distinct Molecular Signatures

**DOI:** 10.1200/GO.22.11000

**Published:** 2022-05-05

**Authors:** Radhika Poojari, Shibashish Giri

**Affiliations:** ^1^ Department of Biosciences and Bioengineering, Indian Institute of Technology Bombay, Mumbai, India; ^2^ Cell Techniques and Applied Stem Cell Biology, Center for Biotechnology and Biomedicine, University of Leipzig, Leipzig, Germany

## Abstract

Genomics landscape in liver cancer is one of the most revolutionizing platforms in the era of next generation sequencing (NGS). Understanding the intra- and inter-liver cancer heterogeneity has an important role in treatment decision-making. Herein, we present a whole transcriptomic based NGS with distinct molecular signatures in metastatic liver cancer tissues of orthotopic SCID mice.

**METHODS:**

High throughput whole transcriptome sequencing was carried out using Illumina platform in ex vivo metastatic liver cancer tissues of orthotopic SCID mice. Healthy normal liver tissues were also subjected to NGS analysis. Pathway analysis was performed using all differentially expressed transcripts.

**RESULTS:**

Approximately 35.4 million sequencing reads and approximately 28.2 million mapped reads were obtained from metastatic liver cancer tissue samples sequenced. We identified approximately 40,018 differently expressed genes. Distinct top 50 significantly differentially expressed transcripts expressed were observed. About 3,840 novel isoforms were identified. The mapping of the differentially expressed transcripts represented major metabolic pathways, immune-related pathways, cell growth-related pathways, the genes involved in metabolism, genetic information processing, and cellular processes.

**CONCLUSION:**

It gives an insight into a better understanding of the molecular mechanisms relevant to liver tumorigenesis. The unique whole transcriptomic molecular signatures could provide an important data to predict the treatment response to molecular therapies in metastatic liver cancer.

